# Seroprevalence of Pandemic H1N1 Antibody among Health Care Workers in Hong Kong Following Receipt of Monovalent 2009 H1N1 Influenza Vaccine

**DOI:** 10.1371/journal.pone.0027169

**Published:** 2011-11-10

**Authors:** Ying Zhou, Diane M. W. Ng, Wing-Hong Seto, Dennis K. M. Ip, Henry K. H. Kwok, Edward S. K. Ma, Sophia Ng, Lincoln L. H. Lau, J. S. Malik Peiris, Benjamin J. Cowling

**Affiliations:** 1 Infectious Disease Epidemiology Group, School of Public Health, The University of Hong Kong, Hong Kong Special Administrative Region, China; 2 Hospital Authority, Hong Kong Special Administrative Region, China; 3 Department of Microbiology, The University of Hong Kong, Hong Kong Special Administrative Region, China; 4 HKU-Pasteur Research Centre, Hong Kong Special Administrative Region, China; National Institutes of Health, United States of America

## Abstract

**Background:**

Healthcare workers in many countries are recommended to receive influenza vaccine to protect themselves as well as patients. A monovalent H1N1 vaccine became available in Hong Kong in December 2009 and around 10% of local healthcare workers had received the vaccine by February 2010.

**Methods:**

We conducted a cross-sectional study of the prevalence of antibody to pandemic (H1N1) 2009 among HCWs in Hong Kong in February–March 2010 following the first pandemic wave and the pH1N1 vaccination campaign. In this study we focus on the subset of healthcare workers who reported receipt of non-adjuvanted monovalent 2009 H1N1 vaccine (Panenza, Sanofi Pasteur). Sera collected from HCWs were tested for antibody against the pH1N1 virus by hemagglutination inhibition (HI) and viral neutralization (VN) assays.

**Results:**

We enrolled 703 HCWs. Among 104 HCWs who reported receipt of pH1N1 vaccine, 54% (95% confidence interval (CI): 44%–63%) had antibody titer ≥1∶40 by HI and 42% (95% CI: 33%–52%) had antibody titer ≥1∶40 by VN. The proportion of HCWs with antibody titer ≥1∶40 by HI and VN significantly decreased with age, and the proportion with antibody titer ≥1∶40 by VN was marginally significantly lower among HCWs who reported prior receipt of 2007–08 seasonal influenza vaccine (odds ratio: 0.43; 95% CI: 0.19–1.00). After adjustment for age, the effect of prior seasonal vaccine receipt was not statistically significant.

**Conclusions:**

Our findings suggest that monovalent H1N1 vaccine may have had suboptimal immunogenicity in HCWs in Hong Kong. Larger studies are required to confirm whether influenza vaccine maintains high efficacy and effectiveness in HCWs.

## Introduction

In 2009 the first influenza pandemic of the 21^st^ Century was associated with a novel influenza A(H1N1) virus that emerged in North America and rapidly spread around the world [1]. In Hong Kong, the first imported pH1N1 case arrived in Hong Kong on April 30, 2009. Activity of pH1N1 peaked locally in September and had subsided by November [2]. pH1N1 was a notifiable condition throughout the first wave and 36,000 laboratory-confirmed cases were notified including 1,400 HCWs, from a local population of 7 million including 150,000 HCWs [2]–[4]. Vaccination is considered as the most effective preventive measure and a series of studies found that one dose in adults and two doses in children of monovalent pH1N1 vaccine were sufficient to generate seroprotective levels of antibody against pH1N1 [5]–[10]. The Hong Kong government purchased 3 million doses of the Panenza monovalent pH1N1 vaccine manufactured by Sanofi Pasteur, which was the only pH1N1 vaccine formulation used in Hong Kong. Local health authorities started to administer the vaccine to members of five target groups including HCWs from December 21, 2009, and extended the vaccination campaign to the general community in January 2010 [11].

We conducted a cross-sectional study of the seroprevalence of pH1N1 antibody among HCWs in Hong Kong following the first epidemic wave. In a separate study we investigated antibody seroprevalence in HCWs who reported that they had not received pH1N1 vaccine [4]. In this study we focus on HCWs who reported receipt of pH1N1 vaccine and investigate factors associated with antibody seroprevalence following vaccination.

## Methods

### Study design

We recruited HCWs between February 11 and March 31, 2010 in 6 public hospitals comprising the Hong Kong West cluster of the local Hospital Authority, with a total workforce of around 7,000 HCWs in one acute care teaching hospital and five non-acute hospitals [4]. We established fixed study locations in each hospital, and participants were invited to participate in our study by open advertisement to all cluster employees. Some participants were approached for recruitment during their regular health check in the cluster staff clinic. HCWs were eligible to participate if they were Hong Kong residents and had worked in the cluster for at least one month. Subjects provided 3 ml of clotted blood, and other information including subject characteristics, history of exposure to influenza infection, and pandemic and seasonal vaccination history was collected by trained research assistants on a short questionnaire. The study protocol was approved by the Institutional Review Board of the University of Hong Kong/Hospital Authority Hong Kong West Cluster. Written informed consent was obtained from all participants.

### Laboratory methods

Serum samples were stored in a refrigerated container at 2–8°C immediately after collection and delivered to the laboratory at the end of each working day for storage at −70°C prior to testing. Serum specimens were tested for antibody responses to A/California/04/2009 (H1N1) by hemagglutination inhibition (HI) and viral microneutralization (VN) assays using standard methods as previously described [4], [12], [13]. The hemagglutination inhibition (HI) test was carried out in 96 well microtitre plates using reagents provided by World Health Organization (WHO) Collaborating Centre for Reference and Research on Influenza Melbourne or the WHO Collaborating Centre, Centres of Disease Control, Atlanta, GA using standard methods as detailed in the WHO reagent kit and elsewhere. The pandemic H1N1 HA antigen was not included in the WHO reagent kit and was prepared by culture of A/California/04/2009 (H1N1) virus in MDCK cells.

The conventional neutralization test for the A/California/04/2009 was carried out in micro-titre plates using neutralization of virus cytopathogenic effect (CPE) in Madin-Darby Canine Kidney (MDCK) cells. Serial serum dilutions in quadruplicate were mixed with 100 tissue culture infectious dose 50 (TCID50) for 2 hours and added to MDCK cells. One hour after infection, serum-virus mixtures were removed and serum free MEM with 2 ug/ml trypsin was added to each well. The plates were incubated and cytopathic effect was observed to determine the highest serum dilution that neutralized ≥50% of the wells. A virus back titration and positive and negative control sera were included in each assay.

### Statistical analysis

We compared the differences in the proportion of HCWs with pH1N1 antibody titer ≥1∶40 both by HI and VN between groups with chi-squared tests or Fisher's exact test and used the phi coefficient to compare results between the two assays. We compared antibody seroprevalence over time in our study with the date of administration of pH1N1 vaccines to HCWs in Hong Kong [14], [15]. We used Generalized Estimating Equation model to estimate 95% confidence intervals for seroprevalence adjusting for potential clustering within hospitals. Among HCWs who did not report confirmed pH1N1 infection, we used univariable and multivariable logistic regression to explore associations between presence of pH1N1 antibody titer ≥1∶40 by HI and VN versus the following factors: sex, age, and seasonal vaccine history in the previous three years. Factors with p-value of 0.2 or lower in univariable analyses were included in the multivariable models. Multiple imputation was used to allow for a small amount of missing data on some background characteristics and make the most use of all available data [16].

## Results

We recruited 703 HCWs during the study period, and seroprevalence data for the 599 HCWs who reported that they had not received pH1N1 vaccine have been reported elsewhere [4]. Among the 104 HCWs who reported receipt of pH1N1 vaccine and who are the focus of the following analyses, 56 (53.8%, 95% CI: 44.2%–63.2%) had antibody titer ≥1∶40 by HI and 44 (42.3%, 95% CI: 33.2%–52.0%) had antibody titer ≥1∶40 by VN. The phi coefficient for the comparison of antibody titers ≥1∶40 by HI or VN was 0.75.

**Figure 1:**
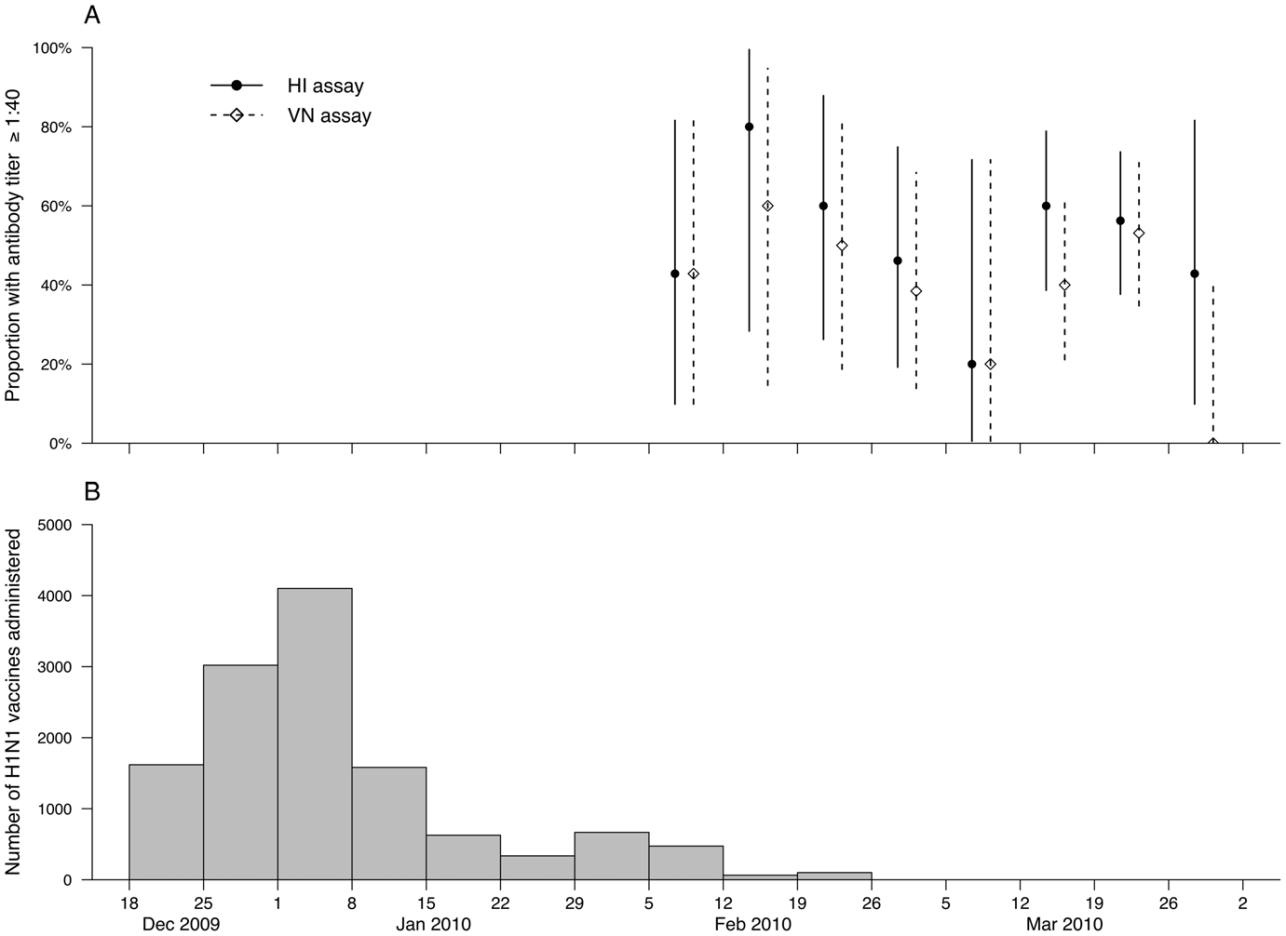
Timing of local vaccination campaign versus serum collection in our study. (A) Among 104 healthcare workers who reported receipt of pandemic (H1N1) 2009 vaccine, the proportion of healthcare workers with antibody titer ≥1∶40 to pandemic (H1N1) 2009 by hemagglutination inhibition (HI) or viral microneutralization (VN) by week of specimen collection with 95% confidence intervals; (B) the number of pandemic (H1N1) 2009 vaccines administered to 150,000 healthcare workers in Hong Kong from December 2009 through April 2010 (source: Department of Health, Hong Kong [14], [15]).

Around 10% of HCWs in Hong Kong had received pH1N1 vaccine by January 2010 (Figure 1). We found no evidence of an increase or decrease in antibody seroprevalence over time in participants, and most participants were recruited more than a month after the vaccination campaign had halted (Figure 1). Three HCWs (46/M, 20/F, 55/F) reported having had confirmed pH1N1 infection by reverse transcription polymerase chain reaction [3] in addition to receiving pH1N1 vaccine, and 2/3 had antibody titer ≥1∶40 both by HI and VN while the other subject had antibody titer <1∶40 by both assays.

Among the 101 HCWs without confirmed pH1N1 infection, the proportion of HCWs with antibody titer ≥1∶40 by VN significantly decreased with older age (test for trend, p<0.001), and was significantly lower among HCWs who reported receipt of 2007–08 seasonal influenza vaccine (Table 1). In the logistic regression model, statistically significant differences remained by age while no statistically significant differences remained by seasonal influenza vaccine history (Table 2). There was also a statistically significant difference in the proportion of HCWs with antibody titer ≥1∶40 by HI among age groups, but not by seasonal vaccine history (Table 1).

**Table 1:** Characteristics of 101 healthcare workers who reported receipt of monovalent pandemic (H1N1) 2009 influenza vaccine and did not report confirmed pH1N1 infection.

Characteristic^a^	No.	Proportion with antibody titer ≥1∶40 by HI (95% CI)		p-value^b^	Proportion with antibody titer ≥1∶40 by VN (95% CI)		p-value^b^
Age 19–24 years	10	100%	(69%–100%)		90%	(56%–100%)	
25–34 years	16	56%	(30%–80%)		50%	(25%–75%)	
35–44 years	25	52%	(31%–72%)		44%	(24%–65%)	
45–54 years	32	44%	(26%–62%)		31%	(16%–50%)	
55–64 years	18	44%	(22%–69%)	0.02	22%	(6.4%–48%)	<0.01
Female sex	75	52%	(40%–64%)		39%	(28%–51%)	
Male sex	26	58%	(37%–77%)	0.78	50%	(30%–70%)	0.44
Suffered febrile influenza-like illness^c^ since July 2009							
No	40	53%	(36%–69%)		35%	(21%–52%)	
Yes	61	54%	(41%–67%)	0.96	46%	(33%–59%)	0.38
Receipt of 2009–10 seasonal influenza vaccine							
No	31	61%	(42%–78%)		52%	(33%–70%)	
Yes	69	51%	(38%–63%)	0.66	38%	(26%–50%)	0.28
Receipt of 2008–09 seasonal influenza vaccine							
No	42	60%	(43%–74%)		52%	(36%–68%)	
Yes	57	49%	(36%–63%)	0.41	33%	(21%–47%)	0.09
Receipt of 2007–08 seasonal influenza vaccine							
No	38	61%	(43%–76%)		53%	(36%–69%)	
Yes	59	48%	(34%–61%)	0.29	32%	(21%–46%)	0.07

a Some columns do not total 104 due to missing data.

b p-values for association calculated by chi-squared tests or Fisher's exact tests.

c influenza like illness is defined as temperature ≥37.8°C plus cough or sore throat.

**Table 2:** Univariable and multivariable analysis of the factors associated with antibody titer ≥1∶40 to pandemic (H1N1) 2009 by viral neutralization among 101 healthcare workers who reported receipt of pandemic (H1N1) 2009 vaccine.

Characteristic^a^	Crude odds ratio of titer ≥1∶40 (95% CI)		Model 1^b^		Model 2^c^		Model 3^d^	
			Adjusted odds ratio of titer ≥1∶40 (95% CI)		Adjusted odds ratio of titer ≥1∶40 (95% CI)		Adjusted odds ratio of titer ≥1∶40 (95% CI)	
Age, years								
19–24	11.45	(1.25–104.60)	15.58	(1.63–149.34)	10.89	(1.16–102.07)	14.08	(1.39–142.27)
25–34	1.27	(0.36–4.48)	1.51	(0.41–5.53)	1.42	(0.39–5.14)	1.48	(0.41–5.40)
35–44	1.00		1.00		1.00		1.00	
45–54	0.58	(0.20–1.72)	0.76	(0.24–2.38)	0.75	(0.24–2.37)	0.74	(0.23–2.34)
55–64	0.36	(0.09–1.42)	0.52	(0.13–2.17)	0.36	(0.08–1.67)	0.39	(0.08–1.82)
Did not receive seasonal influenza vaccine in 2008–09	1.00		1.00				1.00	
Received seasonal influenza vaccine in 2008–09	0.46	(0.20–1.04)	0.45	(0.18–1.11)			0.49	(0.13–1.84)
Did not receive seasonal influenza vaccine in 2007–08	1.00				1.00		1.00	
Received seasonal influenza vaccine in 2007–08	0.43	(0.19–1.00)			0.61	(0.24–1.55)	1.05	(0.27–4.07)

a Multiple imputation was used to adjust for a small amount of missing data on some characteristics.

b Model 1: adjusted for the variables of age group and vaccination history in 2008–09.

c Model 2: adjusted for the variables of age group and vaccination history in 2007–08.

d Model 3: adjusted for the variables that had p-value<0.2 in univariable analyses, i.e. age group, vaccination history in 2007–08, and vaccination history in 2008–09.

## Discussion

Influenza vaccination is recommended as the primary prevention measure against infection, and HCWs are often one of the groups targeted to receive vaccine not only for their direct protection but also to indirectly protect vulnerable patients against nosocomial infection [17], [18]. However seasonal influenza vaccine uptake rates are low among HCWs in many countries [19]–[24], and pH1N1 vaccine uptake was also low in Hong Kong following intense media coverage of a series of adverse events potentially associated with pH1N1 vaccine [11], [14], [15]. Mandatory influenza vaccination policies for HCWs remain controversial [18], [25]–[27]. Around 15% of HCWs in our study reported receipt of one dose of pH1N1 vaccine, compared to overall vaccine coverage of around 10% of HCWs in Hong Kong. However, among HCWs who reported receipt of pH1N1 vaccine, only 54% had antibody titers at or above the 1∶40 level by HI that is conventionally associated with protection against infection [28]. Among 599 HCWs who did not report receipt of pH1N1 vaccine, 12% had antibody titer ≥1∶40 by VN during the same time period [4], which was much lower than those who reported receipt of the pH1N1 vaccine. In two studies of non-adjuvanted monovalent inactivated pH1N1 vaccine from the same manufacturer, 93% and 98% of adults achieved antibody titer ≥1∶40 by HI within 21 days [29] and immunogenicity appeared similarly high for other non-adjuvanted pH1N1 vaccines [5], [6], [8]. Thus the monovalent pH1N1 vaccine appears to have had only moderate immunogenicity in HCWs in Hong Kong. There are three potential explanations for our findings. Firstly, our results may indicate truly poor immunogenicity of the vaccine specifically in HCWs who have had more repeated annual seasonal influenza vaccine compared with general population; secondly, our results may reflect imperfect effectiveness of influenza vaccine in field condition for all recipients of the vaccine; thirdly, our observed results may be biased by delays from vaccination to serum collection.

While only 42% of HCWs had antibody titers ≥1∶40 by VN, which is consistent with the findings of another study of 409 HCWs in Japan who were provided with one dose of pH1N1 vaccine, and just 38% achieved antibody titers ≥1∶40 by HI within 21 days of vaccination [30], 90% of confirmed cases achieved this titer within 3–4 weeks of infection using the same assay in the same laboratory [31]. We previously estimated that around 10%–15% of HCWs were infected through the first wave and cumulative incidence of infection was higher in younger HCWs [4] although the majority of infections were not confirmed by laboratory testing [3]. Higher pH1N1 antibody seroprevalence in younger HCWs who reported receipt of pH1N1 vaccine (Table 1) could be associated with a greater risk of infection rather than better immunogenicity [4], [12]. In addition, reduced immunogenicity of pH1N1 vaccine in older adults has been reported in some previous studies [5], [6], [8], [10]. The proportion of HCWs with antibody titer ≥1∶40 by VN was significantly lower among HCWs who reported receipt of 2007–08 seasonal influenza vaccine (Table 1), while no statistically significant differences remained by seasonal influenza vaccine history after adjustment (Table 2). An early report of the immunogenicity of the same pH1N1 vaccine used in Hong Kong found that geometric mean titer rises between baseline and 21 days after vaccination were significantly reduced in adults who had previously received seasonal influenza vaccines in one or more years between 2004–05 and 2007–08, but the geometric mean titer was not associated with receipt of seasonal vaccine in 2008–09 [10]. Lowered antibody response to pH1N1 vaccine was also associated with prior receipt of 2009–10 seasonal vaccine among pregnant women in Japan [32], among infants and children in Australia [33] and among high school students in Korea [34]. Pre- or co-vaccination with seasonal influenza vaccine was associated with poorer immunogenicity of a pH1N1 vaccine in two randomized trials [35], [36], while pre- or co-vaccination with pH1N1 vaccine was not found to affect the immunogenicity of a seasonal influenza vaccine [36]. In addition to the association between seasonal vaccine and pandemic vaccine, significant association within seasonal vaccines in various years was also supported by some studies. Poorer immunogenicity in subjects with history of prior seasonal influenza vaccine was also reported in studies of a seasonal A(H3N2) vaccine in adults [37], an adjuvanted pH1N1 vaccine in adults [38] and an adjuvanted influenza A(H5N1) vaccine in children [39]. Repeat vaccination with the same strains has also been associated with poorer immunogenicity of seasonal influenza vaccines [40], [41]. Biological explanations for the phenomenon of reduced immunogenicity associated with repeated influenza vaccinations remain unclear [30], [32], [36].

Another possible reason for lower antibody response is imperfect effectiveness of vaccine in field condition for the general population. Various controlled trials have shown good immunogenicity of the pH1N1 vaccine [5], [6], [8], [29]. To be effective in the field, however, a number of elements in vaccination programs need to be implemented properly, including proper cold chain and vaccine management, at all levels; careful logistics management; and safe management of the waste created during the campaign. Failure to properly implement these can reduce the level of protection that is expected from a vaccination program. A sero-epidemiological survey in UK showed that proportion with HI titers ≥1∶32 in the 1–4 years old age-band started to decline after receipt of monovalent vaccine [42], although increases in seroprevalence would be expected after vaccine was being delivered from January 2010 onwards for all children. In Hong Kong, influenza vaccines are distributed every year through the Hospital Authority staff clinics and special vaccination clinics, and the Hospital Authority has substantial experience in maintaining the cold chain and properly administering the vaccine. A separate study in Hong Kong found overall seroprotection rates determined by conventional MN and HI assays in vaccine recipients were 44.8% and 35.1% respectively within 243 days of vaccination [43]. Although information was not provided on the average delays between vaccination and serum collection in that study, those results could also be consistent with somewhat poorer pH1N1 vaccine immunogenicity in the general population in Hong Kong.

Although we did not record time of receipt of pH1N1 vaccine in participants in our study, the majority of HCWs in Hong Kong had received pH1N1 vaccine before mid-January 2010, i.e. at least one month before the start of our study (Figure 1). Antibody response induced by pH1N1 vaccine should be detectable within 21 days of receipt [5], [6], [8], [9], and there was no indication that the proportion of HCWs with antibody titers ≥1∶40 by HI or VN changed over time (Figure 1). Seroprotection rates following seasonal influenza vaccination do not tend to drop substantially from their peak, within 3 months of vaccination [44], [45]. Another study found that geometric mean titers could remain above seroprotective levels within 3 months of pH1N1 vaccination for adults aged between 18 and 28 [46]. Therefore a delay or decline in antibody response following receipt of pH1N1 vaccine is unlikely to explain our findings.

It is important to note several limitations of our study in addition to the relatively small sample size. First, we conducted a cross-sectional seroprevalence study following the first pH1N1 wave, and we did not have baseline (pre-pandemic) data to enable us to compare the pre-pandemic antibody titer with the post-pandemic antibody titer among HCWs [4]. Second, the 104 participants in our study were a small convenience sample covering HCWs in both acute and non-acute hospitals, while a random larger sample would have been ideal albeit more difficult to implement with a high response rate. Previous vaccination history and exposure assessment was based on self-report and recall bias may have influenced the results. HCWs who were more concerned about their health status in the pandemic might have been more willing to participate in our study than others, while also having protected themselves better when caring for patients and been more willing to receive influenza vaccines. Finally, we recruited HCWs who were working in 6 public hospitals on Hong Kong island and our results may not generalize to HCWs working in other regions of Hong Kong or local private hospitals and outpatient clinics.

Vaccination is the most effective way to protect HCWs and the patients around them from influenza, but uptake of influenza vaccines tends to be low in many countries including Hong Kong. Our study indicated lower antibody response than expected among HCWs after receipt of pH1N1 vaccine than antibody results of other two clinical studies using the same pH1N1 vaccine [29]. Further studies are recommended to confirm the immunogenicity of the monovalent pH1N1 vaccines used in 2009–10, and to investigate whether influenza vaccine retains high efficacy and effectiveness in HCWs, many of whom receive annual influenza vaccination, compared to other healthy adults with less prior vaccine experience.
